# Effectiveness of Harm Reduction Interventions in Chemsex: A Systematic Review

**DOI:** 10.3390/healthcare12141411

**Published:** 2024-07-15

**Authors:** Pablo Del Pozo-Herce, Antonio Martínez-Sabater, Paula Sanchez-Palomares, Paula Cristina Garcia-Boaventura, Elena Chover-Sierra, Raquel Martínez-Pascual, Vicente Gea-Caballero, Carles Saus-Ortega, María Luisa Ballestar-Tarín, Piotr Karniej, Enrique Baca-García, Raúl Juárez-Vela

**Affiliations:** 1 Department of Psychiatry, Fundación Jiménez Díaz University Hospital, 28040 Madrid, Spain; pablo.pozo@quironsalud.es (P.D.P.-H.); ebaca@quironsalud.es (E.B.-G.); 2 Instituto de Investigación Sanitaria de la Fundación Jiménez Díaz, 28040 Madrid, Spain; 3 School of Nursing, Fundación Jiménez Díaz, Madrid Autonomous University, 28049 Madrid, Spain; paulac.garcia@estudiante.uam.es; 4 Nursing Care and Education Research Group (GRIECE), GIUV2019-456, Nursing Department, Facultat d’Infermeria i Podologia, University of Valencia, 46010 Valencia, Spain; antonio.martinez-sabater@uv.es (A.M.-S.); ramarpa3@alumni.uv.es (R.M.-P.); m.luisa.ballestar@uv.es (M.L.B.-T.); 5 Care Research Group (INCLIVA), Hospital Clínico Universitario de Valencia, 46010 Valencia, Spain; 6 Nursing Department, Jaume I University, 12006 Castellón, Spain; al414129@uji.es; 7 Internal Medicine, Consorci Hospital Universitari de Valencia, 46014 Valencia, Spain; 8 Research Group Community Health and Care, Faculty of Health Sciences, International University of Valencia, 46002 Valencia, Spain; vagea@universidadviu.com; 9 Nursing School La Fe, Adscript Centre, University of Valencia, 46026 Valencia, Spain; 10 Research Group GREIACC, Health Research Institute La Fe, 46016 Valencia, Spain; 11 Faculty of Finance and Management, WSB MERITO University in Wroclaw, 53-609 Wroclaw, Poland; piotr@karniej.pl; 12 Research Group in Care, Department of Nursing, Faculty of Health Sciences, University of La Rioja, 26006 Logroño, Spain; raul.juarez@unirioja.es

**Keywords:** systematic review, chemsex, harm reduction, risk behavior, health promotion

## Abstract

The phenomenon of chemsex has emerged as an essential public health issue in recent years. This systematic review aimed to investigate currently available harm reduction strategies and to evaluate the efficacy of the corresponding interventions. Methods: A systematic review of the scientific literature related to harm and risk reduction strategies and the effectiveness of chemsex interventions. Between January 2024 and May 2024, the articles were retrieved from the electronic databases Pubmed, Web of Science, Scopus, PsycInfo, Cochrane, Dialnet, CUIDEN, and SciELO. The review protocol was registered in the PROSPERO database (registration number CRD42024508953). The inclusion criteria were as follows: (I) original studies published in peer-reviewed journals, (II) studies exploring harm reduction interventions for chemsex, and (III) studies reflecting the efficacy of harm reduction interventions for chemsex. Two reviewers independently selected articles by title, abstract, and full paper review and extracted data. Two authors described the selected studies and assessed their methodological quality. Results: The systematic review comprised six scientific papers that met the selection criteria and were obtained from five countries. Although a limited number of studies were included, it was observed that they presented a medium–high methodological quality. Programs evaluated interventions to reduce harm from chemsex, such as a web-based intervention that improved self-efficacy to refuse risky behaviors and accept HIV testing. The studies suggested that peer-led programs can be effective, especially with facilitators who have experienced chemsex dependence. Conclusion: Harm reduction strategies in chemsex are effective and should be promoted by health professionals. Interventions should be accessible, personalized, and non-judgmental to provide appropriate care and support, ensuring a comprehensive and effective public health response.

## 1. Introduction

According to the National Institute on Drug Abuse (NIDA), addiction is characterized by compulsive drug-seeking behavior and use despite harmful consequences [1]. This definition aligns with the DSM-5 classification of substance-related disorders, which categorizes them into distinct classes: alcohol, caffeine, cannabis, hallucinogens (such as phencyclidine, LSD, and similar substances), inhalants, opioids, sedatives, hypnotics or anxiolytics, stimulants (including amphetamine-type substances and cocaine), tobacco, and other unspecified substances [2].

While specific groups of psychoactive substances are well-recognized as potential triggers for substance-related or addictive disorders, it is essential to acknowledge that such disorders can also emerge from the use of other substances or the consumption of unknown substances [3]. In recent years, a notable increase in substance use has been observed among men who have sex with men (MSM) within sexual contexts such as sessions, parties, and other social gatherings [4,5,6,7]. This phenomenon, commonly referred to as chemsex, a term coined and popularized by David Stuart in the United Kingdom [6], is characterized by the intentional use of drugs to prolong sexual encounters among homosexual, bisexual, and other men who have sex with men (MSM), with an emphasis on extended duration as a defining feature [5,8]. The term “Chemsex” is derived from the combination of “chems” (chemical substances) and “sex” [5]. In the case of intravenous substance use, it is known as slam or slamming [9,10]. There is considerable variability in the terminology used to describe the phenomenon of substance use within specific cultural contexts, reflecting the global nature of the issue. For instance, terms such as “Chemsex” are commonly associated with the LGTBIQ+ culture, while in Australia, the term “Intensive sex partying” is prevalent [11].

Similarly, in North America, the term “Party and Play” (PnP) is frequently used to describe similar practices, and the term “sexualized drugs” is more widely recognized in the general population [11].

Patterns of substance consumption and sexual behavior in chemsex are characterized by prolonged sexual episodes, known as “chills”, during which individuals engage in sequential sexual encounters with different partners, either individually or in groups, while consuming substances through various routes of administration, with intravenous use posing the most significant risk [8]. Chemsex represents a growing public health concern, with estimated prevalence rates ranging from 3% to 29% among men who have sex with men [8].

As the duration of chemsex sessions increases, so does the exposure to risks and harms associated with substance use, as well as the risk of infections and sexually transmitted diseases [12]. Mixtures of substances can potentiate and prolong their effects, negatively impacting emotion processing and behavior [13,14]. This phenomenon significantly influences the initiation and progression of substance use. It has a profound impact on the mental health of individuals, posing significant public health, sexual, occupational, and social challenges [14,15,16].

The chemsex phenomenon is closely associated with risky sexual behaviors and an increased incidence of sexually transmitted infections [4,17,18,19,20,21,22]. Practices such as unprotected anal sex (barebacking), fisting, and group sex are common. They are linked to a higher risk of contracting infections such as gonorrhea, chlamydia, syphilis, hepatitis C, and HIV, among others [20,23,24,25]. Additionally, studies have shown that chemsex is associated with the emergence of mental health issues such as anxiety, depression, psychosis risk, suicidal ideation, social isolation, stigmatization, and loss of impulse control [13,26]. Moreover, a lack of awareness about the risks and consequences of substance use can lead to intoxication, drug interactions, mental health problems, accidents, and overdose [5,8,24,27].

Furthermore, the social impact of chemsex should not be overlooked, as it often leads to feelings of shame, guilt, and negative thoughts related to excessive drug use and partying, as well as loss of impulse control and altered behavior [9,10,28]. In the literature, motivations for engaging in chemsex include seeking increased pleasure, disinhibition, managing negative emotions, and even addressing internalized homophobia [29]. Understanding the characteristics of individuals who engage in chemsex is essential, as is identifying any barriers they may face in accessing support services to ensure timely and comprehensive assistance [30].

Similar to other public health challenges, such as injecting drug use, approaches to addressing chemsex include punitive and prohibitive public policies as well as harm reduction strategies. The harm reduction approach encompasses policies, programs, and practices aimed at reducing the adverse health, social, and economic consequences of drug use. Examples of harm reduction initiatives include syringe exchange programs and supervised drug injection rooms [30]. Harm reduction strategies complement prevention efforts [31,32].

This article explores currently available harm reduction strategies and the effectiveness of interventions to clarify their impact, thereby addressing the question: “Are harm reduction strategies for chemsex effective?” By doing so, we aimed to investigate currently available harm reduction strategies for chemsex and to evaluate the efficacy of corresponding interventions.

## 2. Materials and Methods

### 2.1. Search Methods for Eligible Articles

This review followed the Preferred Reporting Items for Systematic Reviews and Meta-Analyses (PRISMA) guidelines [33]. The review protocol was registered in the PROSPERO database (registration number CRD42024508953). A systematic literature search was conducted in PubMed, Web of Science, Scopus, PsycINFO, Cochrane, Dialnet, CUIDEN, and SciELO. There were no date restrictions, and articles were searched in English and Spanish. The last search date was May 2024. The following search terms were used: (chemsex OR “chemsex practices” OR chemsexual) AND (“harm reduction” OR “harm minimization”) AND (interventions OR programs OR strategies) AND (effectiveness OR efficacy OR “outcome assessment”).

### 2.2. Selection Criteria for Identifying Articles

The inclusion criteria for the literature were as follows: (1) Original studies published in peer-reviewed journals. (2) Studies exploring harm reduction interventions for chemsex. (3) Studies reflecting the effectiveness of chemsex harm reduction interventions.

The exclusion criteria for the literature included the following: (1) Studies that do not follow harm reduction recommendations or protocols. (2) Protocols for randomized clinical trials, theoretical studies, and other studies that do not provide measurable results. (3) Reviews.

There were no restrictions by the date of publication. There were also no language restrictions.

### 2.3. Study Selection Process

The articles were selected based on whether they fully or partially answered the research question, fulfilled the inclusion criteria, and had sufficient methodological quality. Titles and abstracts of retrieved papers were independently reviewed by two authors (P.C.G.-B. and P.S.-P.) to identify possible studies that met the inclusion criteria. The two reviewers separately assessed the full text of these potentially eligible studies (P.C.G.-B. and P.S.-P.). Discrepancies between reviews were resolved by discussion, with the participation of a third reviewer (P.D.P.-H.) when necessary. Concordance between reviewers was measured using the Intraclass Correlation Coefficient (ICC).

### 2.4. Quality Assessment

Two authors independently checked the quality of the studies included in the review. Observational studies were analyzed using the STROBE statement with 22 items [34]. Similarly, the quality of randomized clinical trials was analyzed using the CONSORT tool with 25 items [35].

### 2.5. Data Extraction

Two independent authors (P.C.G.-B. and P.S.-P.) identified, verified, and extracted data. The following variables were collected: author, aim, design, country, year of study publication, sample size, type of community intervention, harm reduction strategies, and intervention effectiveness.

The primary outcome we sought was the harm reduction interventions included and the interventions’ efficacy. In addition, we explored which type of interventions led to a more significant reduction in the risk of chemsex practice. Moreover, finally, we evaluated which chemsex kind of practice (slamsex/chemsex) is more related to the risk of mental disorders (psychotic disorder, suicidal behavior, anxiety, depression, etc.).

## 3. Results

**Figure 1:**
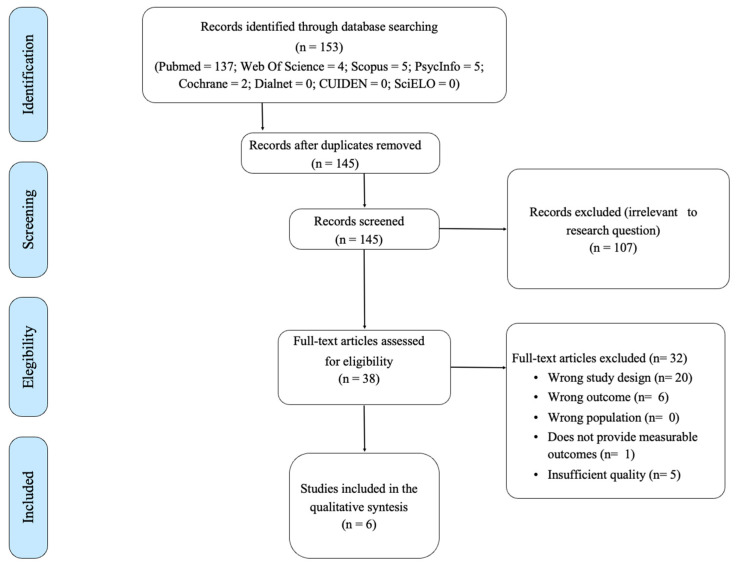
Flow chart of the bibliographical search.

The initial search yielded 153 results. After reviewing the article abstracts and removing duplicate articles, 145 remained. Following a full review and final selection process, six articles were finally included in the review (see Figure 1). The inter-rater reliability (ICC) between reviewers was 0.846 (95% CI 0.54–0.98).

The detailed analysis of the included studies, the characteristics (types of intervention, description of intervention, and tools used), and their effectiveness are shown in Table 1. At the same time, the results (participants, primary outcomes, and quality of the articles are detailed in Table 2. The total sample of studies was 624 individuals, ranging from 29 [36] to 316 [37]. The mean age ranged from 25 [37] to 37 [38]. Of the six definitive articles, one was a cohort study [37], two were a randomized clinical trial [36,39], one was a cross-sectional [40], one was a cross-sectional follow-up study [38], and one was a pilot study [41].

Among the cohort studies, one examined the relationship between the intention to reduce chemsex behavior and chemsex-related mental health service utilization among MSM who engage in chemsex [37]. The other study evaluated the efficacy of an online mindfulness-based cognitive intervention (MBCI) in chemsex MSM [36]. The clinical trial evaluated the efficacy of a web-based intervention to reduce the sexual harms of chemsex among MSM [39]. From the pilot study, the feasibility, retention, and effect of Beyond-66 on chemsex abstinence, abstinence motivation, and mental well-being were examined [41]. A cross-sectional follow-up study evaluates the impact of the training among course participants and participants’ actual performance two years after the course [38]. Another study evaluated the usability and acceptability of a safer chemical package (“PartyPack”) distributed through mobile health devices as a sexual harm reduction strategy among men who have sex with men [40].

The results of the reviewed studies can be classified into approach, interventions, and access difficulties. Regarding approach, Hung et al. [37] highlighted the importance of addressing the lack of knowledge about services available for chemsex and the need to prioritize harm reduction efforts to address this problem. On the other hand, regarding interventions and their effectiveness, it was observed that a web-based intervention focused on harm reduction demonstrated significant improvement in MSM self-efficacy to refuse risky sexual behavior and chemsex, as well as in their acceptance of HIV testing [39]. Mental health clinic attendance was significantly associated with increased intention to reduce chemsex behavior [37] as well as decreased risky sexual behaviors [38] and an increased sense of community responsibility with a knowledge of first aid care [38]. Finally, the pilot study by Thain et al. [41] revealed high completion (76%) and abstinence (74%) rates among participants using Beyond-66, a specific intervention for chemsex-dependent MSM.

In terms of consequences, Hung et al. [37] addressed the stigma associated with chemsex and how this may deter people from seeking help. They indicated that many people who engage in chemsex may not want to change their behavior or use available care services. On the other hand, Thain et al. [41] suggested that peer-led programs may be more effective, especially when facilitators have lived experience of chemsex dependence.

## 4. Discussion

This systematic review aimed to investigate currently available harm reduction strategies and to evaluate the effectiveness of the corresponding interventions. Despite including a limited number of studies, most were of medium to high methodological quality. This analysis was conducted to clarify the influence of these strategies on harm reduction, thus addressing the research question posed.

In recent years, the chemsex phenomenon has emerged as a critical public health challenge. The first mentions in the scientific literature date back to 2015, with several studies dedicated to defining and understanding the concept of chemsex and its relationship with drug use, contact applications, sexually transmitted disease outbreaks, and HIV transmission [18,42,43,44,45]. Recent research underscores the urgency of obtaining reliable and relevant data for a better understanding of chemsex, mainly due to its increasing prevalence, possibly linked to the rise of mobile dating apps [19,46].

Our findings emphasize the importance of investigating the motivations and contexts surrounding chemsex to develop effective risk-reduction measures [7,47]. In our country, studies and government initiatives have been conducted to understand the prevalence and motivations underlying chemsex use [48]. In addition, it has been observed that initial recreational drug use can be a precursor to problematic use [26].

### 4.1. Evaluation of Interventions

The evaluation of community health intervention in a sexual minority population can provide invaluable guidance to health policymakers. A lack of social and family support, along with the stress associated with minority status, may contribute to recreational drug use among men who have sex with men (MSM) and increase the risk of acquiring HIV [49,50]. This assessment can facilitate the design of additional community-focused interventions and the efficient allocation of resources, also allowing for the greater inclusion of community members in policies aimed at risk and harm reduction [38].

Factors such as unemployment, smoking, condomless sex, recent sexually transmitted infections (STIs), the use of post-exposure prophylaxis (PEP), and pre-exposure prophylaxis (PrEP) have been associated with chemsex initiation [49]. Therefore, it is crucial to recognize and support these vulnerable individuals by ensuring equitable access to preventive measures such as PrEP (pre-exposure prophylaxis) for all individuals at risk for HIV [49]. However, there are still regions where the health system does not provide access to PrEP [51].

Collaboration between health services focused on gay, bisexual, other MSM (GBMSM), and drug prevention and recovery services could enhance awareness and access to care [37]. Importantly, chemsex studies should include a comprehensive assessment of individuals, including aspects such as personal situation and social support, to improve risk awareness and facilitate risk reduction measures [49]. Clinical and community-based services focused on the sexual health of MSM are critical for providing education and harm reduction for those who engage in chemsex [52]. The effective integration of these services can result in improved prevention and treatment, promoting the health and well-being of these communities.

In our country, comprehensive care of the chemsex phenomenon has been advocated, involving various actors and care contexts to improve risk reduction according to substances, consumption routes, and sexual practices [8]. Our results underscore the need for a more thorough understanding of chemsex, highlighting the importance of engaging specialized services such as addiction units, sexually transmitted disease clinics, and mental health services. These health facilities can effectively share information and implement chemsex-focused risk prevention campaigns [53].

In this review, we observed that a younger age (<40 years) is associated with a higher likelihood of initiating and dropping out of chemsex [49]. It is critical to individualize care and improve services by measuring and defining behaviors, along with a standardized assessment of outcomes [54]. However, people who engage in chemsex face known barriers in different healthcare settings related to GBMSM care, resulting in the underutilization of health services and worse health outcomes [55,56]. In terms of harm reduction, despite educational strategies and more information being available, chemsex users are perceived differently from other people with dependencies, such as heroin or crack cocaine users, who tend to use addiction care centers where the primary care system has more experience in their management [57]. The MSM perspective is crucial to ensure interventions are tailored to individual contexts, needs, and particularities. It is essential to approach these issues respectfully and sensitively, recognizing and valuing each person’s preferences, needs, and values [58]. However, barriers to consultation in specialized centers persist, mainly due to a fear of being recognized. Therefore, it is crucial to promote existing services, focusing on the areas where chemsex sessions are most frequently organized, and to tailor specific support services for this population [58,59].

In many regions, sexual health, homosexuality, and chemsex are considered embarrassing topics, which can make it challenging to access support services due to stigma, fear of judgment, or concern about the chemsex experience [39,46,58]. In addition, in some countries, there are legal and criminal penalties for substance use, which increases the difficulty of turning to specific care structures and requesting medical and psychological coverage [37,60]. Notably, many men who engage in chemsex may not want to change their behavior or utilize specialized care services, opting instead for harm reduction strategies rather than seeking abstinence [39,44]. Therefore, there is a need for healthcare professionals to generate an awareness of the consequences of sexualized drug use and the availability of harm reduction resources [37]. Perhaps a mindfulness intervention could help raise awareness of drug use by recognizing the triggers. Indeed, Banbury et al. concluded that increasing self-compassion may contribute to controlling emotions such as shame or fear, decreasing the practice of chemsex. Therapies that foster a non-judgmental attitude of acceptance toward beliefs, thoughts, and feelings are crucial for managing shame [36].

The lack of referral options for sexual health professionals seeking to direct clients to facilities with the capacity to provide more in-depth treatment remains a prominent area of improvement for continuity of care [46]. In addition, the lack of harm-reduction guidance from professionals requires synergies between the community and healthcare stakeholders to develop accessible and acceptable harm-reduction strategies [51]. Individuals engaged in chemsex experience moments of both opportunities to change their behavioral health and vulnerability to adverse effects on their well-being. Offering follow-up consultations to address individual needs and provide personalized interventions is crucial. These interventions should be easily accessible according to individual needs, avoiding excessive exposure that may lead to resistance to change or stigmatization [58]. Regarding the characteristics of the interventions, associations have been found between age, PEP use, and taking PrEP with a lower probability of quitting chemsex. On the other hand, being younger than 40 years, being unemployed, having sex without a condom, and having recently had STIs and substance use increase the likelihood of initiating chemsex [39].

### 4.2. Types of Interventions

The urgent need to strengthen screening and care for sexually transmitted infections (STIs) in the MSM population is emphasized [39]. Integrating harm reduction initiatives into health services, psychotherapy, sexology, and addiction counseling services targeting GBMSM is essential. These initiatives include HIV testing and counseling, safe needle/syringe and injection equipment programs, sexual health screenings, and vaccinations [58], along with specific information and education on chemsex and sexual health [37], which are essential. Motivational interviewing and therapeutic education, supported by psychosocial and multidisciplinary options, are critical starting points for addressing chemsex practices, especially in resources- and support facilities-limited settings [58].

Another effective harm reduction strategy is providing chemsex packages through consultations in healthcare settings or mobile health platforms [40]. This initiative aims to support MSM in reducing the harmful effects of chemsex and promoting safe sexual practices. Recognizing the difficulty of quitting drug use abruptly and educating users about safe practices during use can be highly beneficial. The discreet home delivery of these packages ensures marginalized individuals feel safe and less exposed to legal or social repercussions. This discretion also helps alleviate the fear of stigma and discrimination associated with homosexual behavior and chemsex. The applications included in these packages offer confidential and less stigmatizing access to health resources, thereby overcoming barriers to traditional clinical care.

Training courses such as the one by Zucker et al. aimed at combating drug overdoses in gay venues demonstrated a reduction in drug use and sexual risk behaviors among participants. The knowledge, self-empowerment, and confidence gained in the course were associated with increased emergency intervention in gay venues. These results highlight the positive impact of harm reduction initiatives that focus on education and understanding safe recreational drug use rather than outright prohibition or coercive measures. This initiative can be defined as both a harm reduction intervention and a prevention program for a population at high risk of drug overdose [38].

Specific sexual health and harm reduction interventions are required across the health system to address the prevention needs of gay men who combine psychoactive substances with sex. In conclusion, harm reduction strategies for chemsex are effective, and health professionals must promote their implementation. Interventions must be accessible, personalized, and free of bias to ensure that men who engage in chemsex receive appropriate care and support. Additionally, it is essential to combat the stigma associated with chemsex and promote the public awareness of this phenomenon to ensure a comprehensive and effective public health response [21,61,62].

### 4.3. Treatment/Implications for Clinical Practice

The chemsex phenomenon has important implications for clinical practice, as well as for treatment and intervention strategies aimed at reducing the associated harms. In this context, it is crucial to approach this issue from a comprehensive perspective that considers both clinical and psychosocial aspects. Some of the implications and strategies related to chemsex pose significant challenges in contemporary clinical practice. The health implications associated with chemsex are diverse. These range from acute physical complications, such as overdose and injury, to mental health problems, such as depression, anxiety, and substance-induced psychotic disorders, as the practice of chemsex involves the use of recreational drugs such as methamphetamine, mephedrone, and GHB/GBL to enhance sexual experiences. A state of euphoria and disinhibition increases the risk of unprotected sexual practices, which can increase the likelihood of HIV and other STI transmission. In addition, chemsex can negatively affect the adherence to antiretroviral therapy (ART) and PrEP, as the drugs used can alter judgment about reality and awareness, which can lead to missed doses of treatment. Therefore, non-adherence to ART and PrEP can decrease the efficacy of these treatments, exposing individuals to greater health risks [63].

In clinical practice, healthcare professionals must be trained to recognize and appropriately address chemsex regarding diagnosis and treatment. This training implies the need for a comprehensive assessment that includes an exploration of the MSM’s drug use history, sexual health, and mental health. In addition, it is critical to adopt an approach free of prejudice and stigmatization, creating a safe and trusting environment in which people feel comfortable talking openly about their experiences. Regarding treatment, it is essential to consider multidisciplinary approaches that integrate pharmacotherapy, psychotherapy, and harm reduction interventions. Pharmacotherapy can help treat both substance addiction and underlying mental health problems. For example, opioid replacement therapy may be beneficial for those with opioid dependence, while antidepressants may help treat depression associated with chemsex. Psychotherapy, such as cognitive behavioral therapy and acceptance and commitment therapy, can help people develop skills to cope with drug use triggers and improve their emotional health and interpersonal relationships.

In addition to treatment, harm reduction intervention strategies play a crucial role in preventing the harms associated with chemsex. These strategies may include providing accurate information about the health risks associated with drug use, distributing harm reduction kits containing materials for safer use (such as sterile syringes and condoms), and promoting safer sex practices, such as the use of condoms and lubricants.

For all of the above, chemsex poses significant challenges in clinical practice. However, it also offers opportunities to implement effective interventions that address this practice’s medical and psychosocial aspects. By adopting a comprehensive people centered approach, healthcare professionals can significantly reduce the harms associated with chemsex and improve the quality of life of those affected by this practice.

### 4.4. Strengths and Limitations

Several limitations of the present systematic review should be highlighted. First, the search for articles was conducted in English and Spanish, which could have excluded relevant studies published in other languages. Second, methodological heterogeneity among the studies made it difficult to compare the results of the studies included in this review. Third, each study had its criteria for measuring harm reduction strategies and chemsex interventions, as well as study design and inclusion criteria, so the studies may not have accounted for the possibility of confounding variables.

## 5. Conclusions

Our review shows that the results contribute to the evidence that there is an association between the practice of chemsex and the lack of harm reduction strategies and interventions. Risk factors such as age, comorbidity with other pathologies, mental problems, and unemployment have a negative impact on health, as they increase vulnerability to the practice of chemsex and, therefore, to the development of mental disorders that affect the quality of life of individuals. These findings may have clinical implications, so developing chemsex intervention strategies may mitigate harm by reducing STIs and mental problems and improving physical, sexual, and mental health. It would be interesting to continue researching these variables to expand the knowledge of health professionals and strengthen the evaluation and effectiveness of chemsex programs and strategies to prevent its possible consequences. Future research should include a more extensive study sample, as well as the causes, consequences, frequency of use, and subjective and emotional experiences of chemsex users.

## Figures and Tables

**Table 1:** Characteristics of all interventions and analyses.

Author (Year)	Aim	Description of Intervention	Measurement of Outcomes and Variables	Statistical Analyses
Choi et al. (2023) [39]	To evaluate the effectiveness of web-based intervention in reducing the sexual harms of chemsex among MSM.	The intervention consisted of interactive components and knowledge-based information about chemsex in two parts. The first was interactive. Participants completed two questionnaires to assess their understanding of chemsex. Each questionnaire consisted of 10 multiple-choice questions. The second part consisted of educating participants about chemsex, its risks, and legal consequences. Side effects of substances associated with chemsex and information on self-protection against HIV and STIs, as well as local resources for emotional support and testing, were also addressed.	Primary outcomes were assessed with the Chinese version of three instruments. CSES: 14-item instrument with three domains: (1) consistent condom use, (2) correct condom use, and (3) condom use communication. A total score from 14 to 70, a higher score indicates a higher level of condom use efficacy. Cronbach alpha 0.94. SESS: instrument. A 7-item instrument. Total score from 7 to 35, a higher score indicates a higher level of self-efficacy. Cronbach alpha 0.89 DASES: 16 items assessing abstinence self-efficacy across high-risk situations. Total score from 16 to 112, a higher score indicates a higher level of self-efficacy. Cronbach alpha 0.89. All study outcomes were self-assessed at baseline and 3-month follow-up interviews through an online structured questionnaire.	Descriptive statistics were used to summarize the participants’ sociodemographic characteristics and study outcomes at each time point. Baseline characteristics and study outcomes between IG and CG were compared using Fisher exact tests or independent samples t-test. Linear mixed effects models assessed the differential change in continuous outcomes. Generalized linear mixed-effects models with logit links were used to analyze the binary outcomes. The independent variables included time, group, and interaction between group and time.
Hung et al. (2023) [37]	To understand the relationship between the utilization of mental health services among GBMSM who engage in chemsex and the intention to reduce chemsex behavior and use health services.	The chemsex care plan is based on motivational interviewing techniques with regular follow-up visits and prevention and treatment services for persons at risk for HIV/STIs, including testing, counseling, PrEP, and PEP for HIV prevention. Clients were asked to rate the current and ideal proportions of their sexual activities involving the use of MDMA, ketamine, methamphetamine, GHB/GBL, or mephedrone.	Baseline questionnaire during their initial visit in order to collect information on sociodemographic characteristics, substance use, diagnosis of HIV/STIs, PrEP use, and mental health. Follow-up questionnaires were collected during subsequent visits. On a visual analog scale from 0 to 100%, participants were asked to rate the proportion of their sexual activities that involved the use of chemsex. Participants were asked to report whether they had used any substances in the previous year by selecting items from a list of 14 options. The GAD-7 assessment scale was used to evaluate the degree of anxiety, and the PHQ-9 assessment scale was used to evaluate depression.	Descriptive statistics were computed to demonstrate the frequency and distribution of the occurrence of the various participant characteristics. Chi-squared tests were used to compare participants in terms of the intention to reduce chemsex behavior. Logistic and Poisson regressions were used to test for relationships between the use of healthcare services and all other variables. Variables with a *p*-value of less than 0.05 in the univariable models were then entered into the multivariable regression models, along with age and monthly income.
Thain et al. (2024) [41]	To evaluate the feasibility, retention, and effect of Beyond-66 on abstinence from chemsex, motivation for abstinence, and mental well-being.	A pilot evaluation of a novel peer-led harm reduction intervention for chemsex. Demographic characteristics, retention, completion, and abstinence were collected between January 2021 and August 2023 in MSM using Beyond-66. Motivation to remain abstinent and mental well-being at baseline and the end of Beyond-66 were compared using a 10-point Likert scale.	Demographics of the MSM referred to Beyond-66 were collected data on entry into the program, completion, and remaining abstinent at their final review between January 2021 and August 2023. With a 10-point Likert scale, participants were routinely asked to provide a score of how motivated they felt to enter the program; at the end, they were asked to repeat the score, focusing on their motivation to remain abstinent. Participants were asked to score their mental well-being using a 10-point Likert scale at the program’s beginning and end.	Descriptive statistics were used to summarize the sociodemographic characteristics and study outcomes. The Kruskal–Wallis test was used to compare pre- and post-program Likert scores.
Zucker et al. (2022) [38]	To assess the impact of the training among the course participants and to evaluate the participants’ actual performances (two years after the course).	The PK course is a unique 4-hour training course that provides participants with tools to identify, prevent, and treat common medical syndromes associated with substance overuse. The course was offered to MSM and transgender individuals, who were asked to enroll in the training free of charge between January and March 2021.	Anonymous online questionnaire about demographic characteristics, drug use habits before and after the course, personal experience with eight common chemsex and party substances (marijuana, GHB, MDMA, ketamine, cocaine, and methamphetamine), alcohol use, incidence of drug-impaired sex, use of PrEP, and whether they had ever needed emergency treatment for excessive drug use. To assess the effect of the course on participants, a 5-point Likert scale (from “Not at all” = 1 to “Very much” = 5) was used based on eight measures of knowledge and social/community effects: Knowledge acquired in the course (3 items), Awareness of the threat of drug and alcohol use (2 items), Responsibility and trust in the community (3 items). The higher the score, the greater the impact of the course on the participant’s knowledge. Participants were asked whether they had confidence in themselves as PKs and their level of satisfaction with the course (both on a 5-point scale, from “Not at all” = 1 to “To a great extent” = 5) and whether they had had to help people in emergencies at parties or social events after the course. Finally, they were asked to describe a common intervention they had performed as a PK after the course (open-ended question).	The evaluation of the program consisted of several steps. First, the personal impact on PKs was assessed by analyzing changes in their risk behaviors before and after the course using McNemar’s Chi-square test for categorical variables. Secondly, it was assessed how many PKs had assisted people in emergency medical situations at LGBT social events, comparing them with those who had not. Statistical tests such as Chi-square or Student’s t-test were used to compare independent variables, depending on whether they were categorical or continuous. Variables with *p* < 5% in the univariate analyses, in addition to age, were included in the multivariate analysis to identify attributes associated with supporting people in emergencies after the course. Finally, open-ended questions were analyzed using content analysis to provide descriptive statistics of the common interventions of PKs and their need for additional training.
Gautam et al. (2023) [40]	To evaluate the usability and acceptability of a safer chemicals package (“PartyPack”) distributed through mobile health devices as a sexual harm reduction strategy among men who have sex with men.	Intervention based on a smartphone app (JomPrEP) designed to improve access to HIV prevention services among MSM. Participants were informed about downloading the JomPrEP app with instructions on how to use the app to request and track PartyPack for 30 days (between March and April 2022) and complete a post-survey at the end of the study period. They were provided with a single-use registration code needed to access the app. free of charge and only had to provide the mailing address at the location of their choice. Recruitment was conducted through flyers posted in local organizations and social media platforms.	Demographic characteristics: age, ethnicity, educational status, relationship status, income, depressive symptoms, substance use, sexual history, HIV- or STI-testing practices, and past use of PrEP and PEP. Satisfaction with the PartyPack feature was measured with a single-item question: “To what extent are you satisfied with the PartyPack feature of the application?”. A 5-point Likert scale (from 1 = not at all satisfied to 5 = very satisfied) was used. The degree of satisfaction was coded as “satisfied” if the answer was “very satisfied” and “extremely satisfied”. The ease of use of PartyPack was measured with a single-item question: 4-point Likert scale (1 = very difficult to 4 = very easy). Individual interviews were conducted via videoconference with 20 participants (40%) to determine their opinions about Party Pack’s functions, especially its usefulness, preferences for specific elements, and suggestions for improving it.	Means for continuous variables and frequencies for categorical variables were calculated to describe the participants. The usability and acceptability of the PartyPack were based on descriptive statistics from the app analytics and acceptability measure. All exit interviews were audio-recorded, transcribed, and analyzed for qualitative data. The comments and issues were grouped and categorized according to common themes relative to specific app functions by two coders.
Banbury et al. (2023) [36]	To evaluate the efficacy of an online mindfulness-based cognitive intervention (MBCI) in MSM who practice chemsex.	The intervention included 15 domains, in which 35 of the 93 BCTs listed in the BCTv1 taxonomy were identified. The main activities included mindfulness, breathing, relaxation techniques, mindfulness of the senses and the body, and understanding the self. This online MBCI contains cognitive, behavioral, and mindfulness factors. Each session included substance use and sexual behavior, working with the inner critic and high-risk situations, sex without drugs and sexual identity and psychosexual well-being, substance use, and self-compassion. Group 1 was the experimental group receiving MBCI, and Group 2 was the deferred control group. Both study groups completed self-report questionnaires throughout the program at weeks 0 (baseline), 8, and 12. Group 1 did not access the materials until week 1 (follow-up). Group 2 did not access the materials until week 8 when the program began. The assessments lasted approximately 20 minutes. Online group mindfulness sessions lasted 2 to 3 hours every two weeks. Group 2 received MBCI at week 8.	Three tools were used to assess levels of cognitive mindfulness, sexual self-efficacy, well-being, and chemsex use. CAMS-R: a 10-item measure with four response categories (1 = rarely/not at all to 4 = almost always). Higher scores indicate higher levels of mindfulness (range 4–40). Cronbach alpha 0.724. The Sexual Self-Efficacy Scale: an 8-item questionnaire comprising four response categories (1 = not at all to 4 = very much). Cronbach’s alpha ranges between 0.58 and 0.74. Scores range from 6 (little to no self-efficacy) to 24 (high self-efficacy)—Cronbach’s alpha is 0.752. SWEMWBS: a 7-item questionnaire with five response categories looking at functioning and aspects of well-being. The response categories range from 1 = none to 5 = all the time. Scores range from 7 to 35, the latter indicating the highest level of well-being—Cronbach’s alpha 0.730. A 19-item questionnaire with four response categories (1 = never to 4 = always) was developed to assess chemsex engagement. Questions 1–4 focus on drug use, 5–7 self-care and self-compassion, 8–10 lifestyle, 11–14 risk behavior, and 15–19 well-being/mental health. Scores ranged between 19 (low chemsex engagement) and 76 (very high chemsex engagement). Cronbach’s alpha 0.708 A focus group was also held during week 12 to hear opinions, general experiences, and program suggestions. The focus groups lasted approximately one hour, with about five participants per group.	The two groups were compared (between-subjects design) on the efficacy of MBCI on chemical sex use, well-being, and self-efficacy at weeks 0, 8, and 12 (within-subjects design). *T*-test comparisons were made between Groups 1 and 2 at Weeks 0, 8, and 12 and between Group 1 at Week 8 and Group 2 at Week 0. A repeated measures ANOVA was conducted to analyze the effect of time on chemsex, mindfulness and cognition, sexual self-efficacy, and well-being.

CSES: Condom self-efficacy scale; SESS: Sexual Safety Self-Efficacy Scale; DASES: Drug Avoidance Self-Efficacy Scale; IG: intervention group; CG: control group; GBMSM: Gay, Bisexual, and other Men who have sex with men; IQR: interquartile range; BCT: behavior change techniques; MBCI: Mindfulness-based cognitive Intervention; SWEMWBS: Short Warwick–Edinburgh Mental Well-being Scale; CAMS-R: Cognitive and Affective Mindfulness Scale-Revised; GAD-7: Generalized Anxiety Disorder scale. PHQ-9: Patient Health Questionnaire-9; PK: Party Keepers; BCTTv1: Change Technique Taxonomy version 1.

**Table 2:** Characteristics and results of included studies.

Author (Year)	Design	Country	Participants	Outcomes	Quality
Choi et al. (2023) [39]	Randomized controlled trial.	Hong Kong	- MSM (n = 316) Mean age: 27.34 (6.77). - IG = 158. - CG = 158. - Sexual orientation: 265 (83.9%) homosexual; 51 (16.1%) bisexual. - Marital status: 172 (54.4%) in a relationship or married; 144 (45.6%) single. - Employment: 199 (63.0%) employed full-time; 117 (37.0%) not employed full-time. - Monthly income: 167 (52.8%) under HKD 20,000; 149 (47.2%) upper HKD 20,000.	The intervention group showed significantly more significant improvement in self-efficacy to refuse risky sexual behavior and chemsex: - CSES: IG: Mean (SD) = 5.91(12.65); CG: Mean (SD) = 1.64(8.69) *p* = 0.002 (time–group interaction: β = 4.52; 95% CI: 2.03–7.02; *p* < 0.001). - SESS: IG: Mean (SD) = 3.49 (6.97); CG: Mean (SD) = 1.40 (4.54) *p* = 0.006 (time–group interaction: β = 2.11; 95% CI: 0.66–3.56; *p* = 0.004). - DASES: IG: Mean (SD) = 9.18 (25.87); CG: Mean (SD) = 2.03 (19.29) *p* = 0.010 (time–group interaction: β = 6.98; 95% CI: 1.75–12.22; *p* = 0.009). - Intention to have chemsex in the last three months: IG: 28 (17.7%); CG: n (%) 29 (18.4%) *p* = 0.99 (time–group interaction: OR = 0.23; 95% CI: 0.10–0.53; *p* = 0.001). - Participation in chemsex: in the last three months: IG: 27 (17.1%); CG: n (%) 24 (15.2%) *p* = 0.76 (time–group interaction: OR = 0.37; 95% CI: 0.18–0.78; *p* = 0.009). - HIV testing in the last three months: IG: 40 (25.3%); CG: 64 (40.5%) *p* = 0.006. - (time–group interaction: OR = 3.08; 95% CI: 1.72–5.54; *p* < 0.001). - Testing for other STIs in the last three months: IG: 27 (17.1%); CG: n (%) 37 (23.4%) *p* = 0.21.	CONSORT 20/25
Hung et al. (2023) [37]	Cohort study	Taiwan	N = 152 GBMSM practicing chemsex (105 (69.1%) of attending HERO program) - Mean age: 28 (IQR 25–34.25). - Monthly income: 81 (53.3%) less than NTD 30,000. - Educational level: 123 (80.9%) with a bachelor’s degree or higher. - Employment: 109 (71.7%) employed. - Marital status: 79 (52.0%) single, 49 (32.2%) with regular partners, 24 (15.8%) with casual partners. - Substance use: 55 (36.2%) multiple drug use in the last 12 months. A total of 25 (16.4%) high frequency of substance use. Living with HIV: 39 (25.7%) Mental Health: 35 (25.0%) symptoms of anxiety, 41 (29.3%) symptoms of depression, 45 (32.1%) episodes of anxiety or depression in the last two weeks. Intended to reduce chemsex behavior: 105 (69.1%). Most prevalent drugs: methamphetamine (48.0%), GHB/GBL (22.4%), MDMA (13.2%), ketamine (8.6%).	- Intention to have chemsex in the past three months: 57 (18.0%). - Actual lifetime participation in chemsex: 84 (26.6%) - Actual participation in chemsex: in the last three months: 51 (16.1%). - HIV testing in the last three months: 104 (32.9%). - Testing for other STIs in the last three months: 64 (20.3%). - Health service utilization ranged from 23.0% for attending chemsex recovery group meetings, 17.1% for visiting a mental health clinic, and 10.5% for using both services. - Intention to reduce chemsex behavior was significantly associated with visiting a mental health clinic (OR = 4.68, *p* < 0.05). However, its association with attending chemsex recovery group meetings was only marginally significant (OR = 2.96, *p* < 0.1). - Participants who had attended chemsex recovery groups were significantly more likely to use methamphetamine, erectile dysfunction medications, and alkyl nitrites but less likely to consume alcohol (*p* < 0.05). - Participants who had visited a mental health clinic were significantly more likely to use methamphetamine, erectile dysfunction medications, and sedatives (*p* < 0.05).	STROBE 19/22
Thain et al. (2024) [41]	Cohort study	Malaysia	MSM (N = 25), with a median duration of chemsex use of 5 years (Range: 4–6), who had finished the Beyond-66 program between 2021 and 2023.	At the time of assessment, 19 (76%) had completed the 132-day program. A total of 3 (12%) had dropped out AND 3 (12%) had been referred for psychiatric evaluation; 12 (48%) were living with HIV. - In total, 14 of 19 (73.7%) participants remained abstinent at the time of assessment, and 5 (26.3%) had relapsed. - Motivation for abstinence: Mean score increased after completing the program: from 7/10 to 9/10 (*p* = 0.04). - Mental health: Mean mental well-being score (Likert score out of 10 where 10 is poor mental health) decreased significantly after completing the program: from 5/10 to 2/10 (*p* = 0.008).	STROBE 18/22
Zucker et al. (2022) [38]	Cross-sectional follow-up study	Israel	- 52 participants (from 130 MSM). - Mean age 37 years (SD = 5.4, range: 27–53). - Sexual orientation: 47 (90.4%) homosexual; 3 (5.8%) bisexual; 2 (3.8%) heterosexual - Marital status: 30 (57.7%) single, 5 (9.6%) monogamous relationship, 17 (32.7%) open relationship.	- A total of 34 participants (65.4%) were very satisfied with the course, only 4 (7.7%) were not. A total of 50 participants (96.2%) said they would recommend the course to friends. - They reduced their use of drugs, mainly cocaine and alcohol, and increased their use of PrEP. Before the course, 21 (40.4%) participants needed emergency help for drugs; afterwards, this was only 2 (3.8%) (*p* < 0.001). A total of 33 (63.5%) participants provided help in emergencies after the course. - There were no significant demographic differences between those who helped and those who did not. However, those who did exhibit greater confidence levels as PKs gained more knowledge in the course and felt a greater sense of community responsibility and self-confidence than those who did not. - In multivariate analysis, high confidence as PKs (*p* = 0.01) and course knowledge (*p* = 0.02) were associated with helping in emergencies.	STROBE 18/22
Gautam et al., 2023 [40]	Cross-sectional study	Malaysia	- Total of 50 participants, mean age 27.9 (SD 5.3). - Of these, 36 (72%) were single; 26 Malay (52%). - Total of 34 (68%) were university graduates - A total of 36 (72%) liveed with others in a house or apartment; 49 (98%) reported having undergone HIV testing, 39 (78%) did so within six months; 26 (52%) reported self-diagnosis of HIV; 5 (10%) reported using PrEP; 47 (94%) reported anal sex; 16 (32%) used a condom; 4 (8%) practiced chemsex; 9 (18%) practiced group sex.	- In total, 43 (86%) ordered PartyPack: 27 (63%) placed multiple orders during the 30 days. - 95% (41/43) satisfied with Party Packs in-app ordering feature. - 91% (39/43) indicated that ordering and tracking was easy. - Four overarching themes emerged from the thematic analysis: Reasons for ordering and not ordering the PartyPack. usability and acceptability of the PartyPack. feedback and suggestions for improvements. considerations for future use.	STROBE 18/22
Banbury et al. (2023) [36]	Randomized controlled trial.	London	- In total, 29/45 MSM who practiced chemsex - Group 1: 15 MSM; Group 2: 14 MSM - Between 18 and 30 years old (16, 55.2%), - White (25, 86.2%); British (26, 89.7%); Single (21, 72.4%), - Chemsex initiated between 18 and 30 years of age (24, 82.8%), which implied a weekly consumption (18, 62.1%) of poliquim while engaging in sex, which had an impact on the consumption of prescription medication (16, 53.3%), including antiviral drugs (6, 20.7%).	- The MBCI proved to be effective, with 79% of participants maintaining the practice during the three-month follow-up and experiencing a reduction in both substance use and sexual risk behaviors. Satisfaction with the program was high, with 79% affirming its importance in managing sexuality-related substance use. Participants also reported being more compassionate with themselves and more aware of their needs, emotions, and feelings. In addition, lower levels of chemsex and higher levels of cognitive mindfulness, sexual self-efficacy, and well-being were observed after the intervention and at the 12-week follow-up. - Of the participants’ responses, 26.1% felt supported, another 26.1% mentioned that it helped them a lot, 21.7% said they felt better, 13.0% felt more confident, and another 13.0% claimed to feel less chemsex-related stress. Regarding the least helpful aspects of the intervention, 63.6% expressed the need for long-term support, and 36.4% required longer follow-up. - Regarding the experience using the MBCI, 31.8% found the experience generally supportive, another 31.8% perceived it as supportive of them and their use, 22.7% described it as fun, and 13.6% found it helpful in reducing drug use. Regarding MBCI support for drug use, of the 25 responses, 32.0% claimed to use drugs more safely, 28.0% to be more careful, 20.0% to respect themselves more, and another 20.0% to use drugs differently. Regarding how this intervention supported sexual well-being, of the 15 respondents, 26.7% reported feeling more sexual without drugs, another 26.7% understood their sexual needs better, another 26.7% experienced better erections with fewer drugs, and 20.0% were more aware during sober sex. - In terms of how MBCI supported overall well-being, of the 52 responses, 26.9% claimed to work with internal shame, 23.1% to be more self-compassionate, 13.5% to better understand themselves and their needs, another 13.5% to continue using drugs but feel more in control, and 11.5% to better understand their emotions and feelings. In addition, 38.9% appreciated that the intervention helped with drug and other problems, 13.9% learned to love themselves, another 13.9% expressed the need for ongoing support, and 8.3% were surprised by the positive effects of the intervention. - Some statistical differences were found between groups 1 and 2: (a) in chemsex engagement at week 0 (*p* < 0.001), 8 (*p* < 0.001), and 12 (*p* < 0.001); (b) in mindfulness and cognition at week 0 (*p* < 0.001) and 12 (*p* < 0.001); (c) in sexual self-efficacy at Week 0 (*p* = 0.026); (d) in well-being between groups at Week 0 (*p* = 0.040) and 12 (*p* = 0.026).	CONSORT 22/25

CSES: Condom self-efficacy scale; SESS: Sexual Safety Self-Efficacy Scale; DASES: Drug Avoidance Self-Efficacy Scale; OR: odds ratio; IG: intervention group; CG: control group; GBMSM: Gay, Bisexual, and other Men who have sex with men; MBCR: Mindfulness-Based Chemsex Recovery; MBCT: Mindfulness-based cognitive therapy.

## Data Availability

Not applicable.
